# Efficacy and Safety of Panobinostat in Relapsed or/and Refractory Multiple Myeloma: Meta Analyses of Clinical Trials and Systematic Review

**DOI:** 10.1038/srep27361

**Published:** 2016-06-07

**Authors:** Jing-di Liu, Chun-yan Sun, Liang Tang, Ying-ying Wu, Qing-yun Wang, Bei Hu, Yu Hu

**Affiliations:** 1 Institute of Hematology, Union Hospital, Tongji Medical College, Huazhong University of Science and Technology, Wuhan, Hubei, China; 2 Collaborative Innovation Center of Hematology, Huazhong University of Science and Technology, Wuhan, Hubei, China

## Abstract

During the past decades, many novel agents have improved response and survival of patients with multiple myeloma. Nevertheless, it remains challenging when they suffer relapsing. Thus, novel therapeutic agents are needed. We aimed to assess the efficacy and safety of a novel agent panobinostat for patients with relapsed or/and refractory MM. A systematic literature review identified studies for clinical trials about panobinostat in patients with relapsed or/and refractory MM. We searched studies published between January 2000 and December 2015 in Pubmed, Ovid, EBSCO and the Cochrane library. Random-effect pooled estimates were calculated for overall response rate and rates of common adverse effects. The results showed 11 clinical trials including 700 patients with relapsed or/and refractory MM treated with panobinostat were identified. The ORR varied between 0.08 and 0.67. Pooled analyses showed the results that the ORR was 0.45 (95% CI: 0.31–0.59, *I*^2^ = 90.5%, *P* = 0.000) for panobinostat combined with any other kind of drugs. The most common Grade3/4 adverse effects were thrombocytopenia, neutropenia, lymphopenia, anemia, diarrhea, fatigue, nausea and so on. In conclusion, based on our analyses, the regimen of panobinostat combining with other agents seems to be well tolerated and efficacious in patients with relapsed or/and refractory MM.

Multiple myeloma (MM) is a malignant neoplasm of plasma cells that accumulate in bone marrow, leading to hypercalcemia, renal failure, anemia , osteolytic bone lesions and so on^1^,^2^. Treatment of MM has radically changed since the introduction of novel agents proteasome inhibitors (PIs) and immunomodulatory drugs (ImiDs) and their combination with conventional chemotherapeutics (CCs). The current treatment is based on a combination of CCs with PIs and/or ImiDs. According to patient age and performance status, this therapy may be followed by stem cell autologous transplantation^3^. These novel agents significantly improved the response rate, progression-free survival, and overall survival over the last decade, and made it easy to control the disease for longer periods of time^4^. Despite these advances, nearly all MM patients ultimately relapse, even those who experience a complete response to initial therapy^5^,^6^,^7^. Management of relapsed MM represents a vital aspect of the overall care for patients with MM and a critical area of ongoing scientific and clinical research. Thus, MM remains an incurable disease and novel treatment approaches that are not only effective but also safe are needed.

During the past few years, several other novel classes of drugs have been shown to have activity in myeloma in early-stage clinical trials, including the histone deacetylase (HDAC) inhibitors, heat shock protein (HSP) inhibitors, mammalian target of rapamycin (mTOR) inhibitors, phosphoinositide 3-kinase (PI3k)/AKT inhibitors, and monoclonal antibodies^8^,^9^. Our meta analyses and systematic review focus on one of the highly potent HDAC inhibitors called panobinostat which has been demonstrated that can affect the growth and survival of MM cells through alteration of epigenetic mechanisms and protein metabolism^10^,^11^ and it has been found to be effective in patients with relapsed/refractory MM with an acceptable toxicity profile. Panobinostat has been approved by the FDA and EMA recently for use in combination with bortezomib and dexamethasone in patients with relapsed/refractory MM who have received ≥2 prior regimens according to the results from the phase III PANORAMA 1 trial in patients with relapsed or relapsed and refractory MM^12^. Our meta analyses focus on the efficacy and safety of panobinostat combined with other agents in patients with relapsed or/and refractory MM.

## Methods

### Search strategies and selection criteria

**Figure 1:**
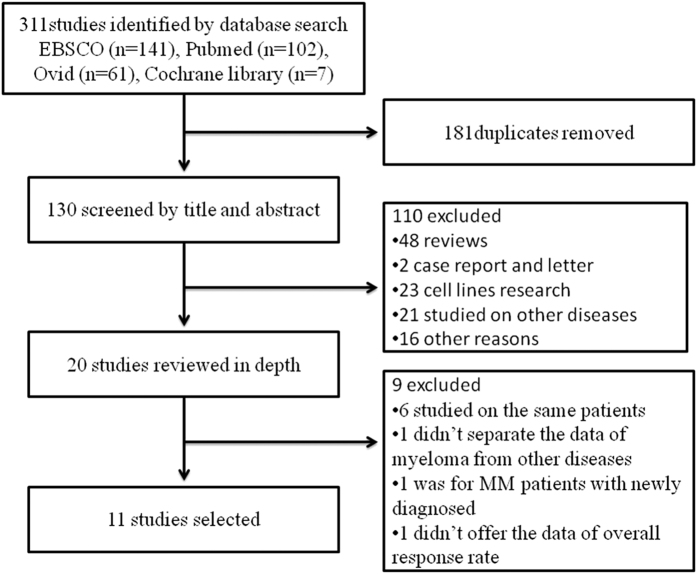
Studies selective strategies.

An electronic search of Pubmed, Ovid, EBSCO and the Cochrane library for clinical trials of Phase I/II/III with terms related with “multiple myeloma”, “myeloma”, “MM”, “panobinostat”, “LBH589”, “pan-deacetylase inhibitor” between January 2000 and December 2015 was performed. All clinical trials were included in our review, no matter what the article types were (whether full-text articles or conference abstracts, whether English or not). Besides, studies were eligible for inclusion in this meta analyses if they met all the following criteria: (i) they were published between January 2000 and December 2015; (ii) the patients who were under studying were relapsed or/and refractory to first-line treatments; (iii) the articles or abstracts showed initial data allowing us to calculate the overall response rate (ORR). In addition, varied articles and/or conference abstracts studied on the same trial at different time points were considered as the same, and only the one with the latest data can be concluded in. The studies were excluded if they met the following criteria: (i) the studies were without initial data such as reviews; (ii) the studies researched in animals or cell lines; (iii) it was a case report; (iv) the studies didn’t provide enough data to let us calculate the ORR. The selective strategies were showed in Fig. 1.

### Outcome measures

The primary objective of our meta analyses was to observe the efficacy of panobinostat combined with other drugs by calculating the overall response rate, clinical benefit rate, rate of stable disease and rate of progressive disease. The second objective was to evaluate the safety by calculating the rate of common adverse effects.

### Data analysis

A random-effects model was applied in all the analyses. We explored ORR (overall response rate), CBR (clinical benefit rate) and rates of SD (stable disease), PD (progressive disease) and AEs (adverse effects) using 95% confidence interval (CI) for panobinostat combined with any agents and with proteasome inhibitor separately. The inter-study heterogeneity was estimated by the Q and I^2^ statistical tests. I^2^ > 50% indicated heterogeneity. If I^2^ was significant (>50%), the random-effects model was selected. Otherwise, the fixed-effects model was selected^13^. All the meta analyses were completed by STATA SE 12.0. A *P*-value < 0.05 was defined as statistically significant for all outcomes.

## Results

### Studies identified

According to our inclusion criteria, 11 clinical trials were identified including a phase III study^14^, 4 phase II studies^15^,^16^,^17^,^18^, 2 phase I/II studies^19^,^20^, and 4 phase I studies^21^,^22^,^23^,^24^. The characteristics and previous treatments of the studies are shown in Tables 1 and 2. 8 studies were trials about ponobinostat combined with proteasome inhibitor (bortezomib or carfilzomib), and 5 of them were combined with both bortezomib and dexamethasone. 1 combined with lenalidomide and dexamethasone, 1 combined with melphalan and 1 combined with melphalan, prednisone and thalidomide. 7 studies showed the detailed data of previous treatments. In total, 700 patients with relapsed or/and refractory multiple myeloma were included in our meta analyses.

### Efficacy

**Figure 2:**
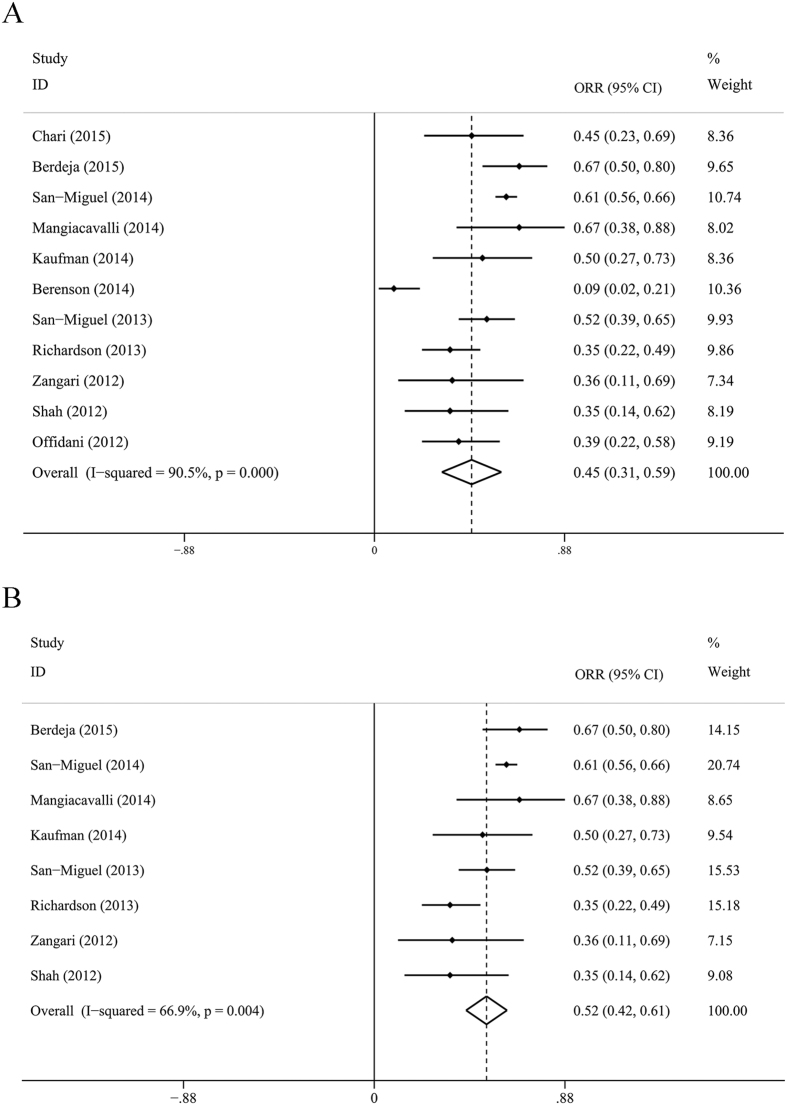
Forest plots for meta analysis of ORR. (**A**) ORR for panobinostat combined with any other drugs (**B**). Subanalysis of ORR for panobinostat combined with proteasome inhibitors with or without dexamethasone. ORR, overall response rate; CI, confidence interval; I-squared values indicate significant heterogeneity between individual studies.

**Figure 3:**
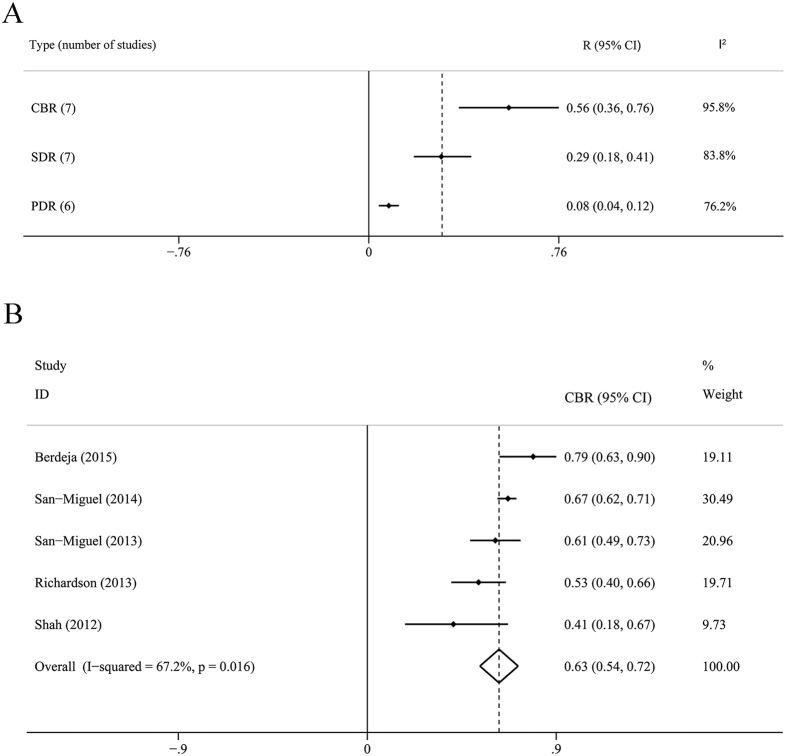
Meta analysis of CBR, SDR, PDR and Subanalysis of CBR. (**A**) Meta analysis of CBR, rates of SDR and PDR (**B**). Subanalysis of CBR for panobinostat combined with proteasome inhibitors with or without dexamethasone. CBR, clinical benefit rate; SDR, rate of stable disease; PDR, rate of progressive disease.

The ORR of all the studies was 0.45 (95% CI: 0.31–0.59, *I*^2^ = 90.5%, *P* = 0.000), and ORR = 0.52 (95% CI: 0.42–0.61, *I*^2^ = 66.9%, *P* = 0.004) for the subanalysis of panobinostat combined with proteasome inhibitor with or without dexamethasone. The ORR was 0.51 (95% CI: 0.39–0.63, *I*^2^ = 74.8%, *P* = 0.003) for another subanalysis of panobinostat combined with bortezomib and dexamethasone. The CBR was 0.56 (0.36–0.76), the SDR was 0.29 (0.18–0.41) and the PDR was 0.08 (0.04–0.12). Detailed results were showed in Figs 2 and 3.

### Adverse effects

**Figure 4:**
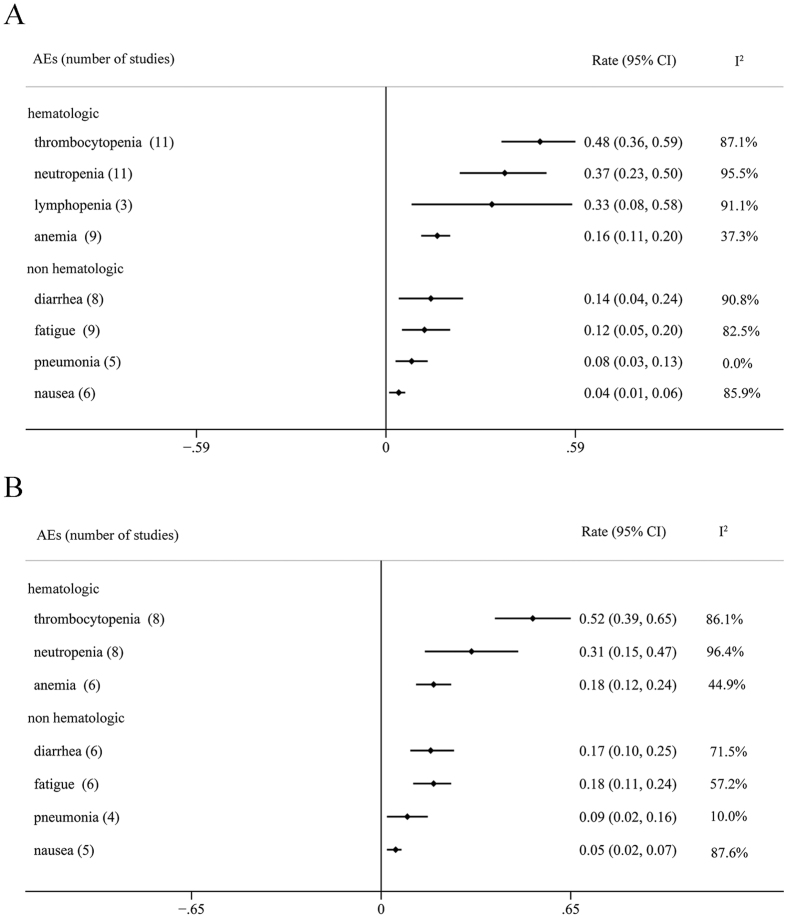
Meta analysis of common AEs. (**A**) Rate of AEs for panobinostat combined with any other drugs (**B**). Subanalysis of Rate of AEs for panobinostat combined with proteasome inhibitors with or without dexamethasone. AE, adverse effects.

The most common Grade 3/4 adverse effects were divided into two groups. The hematological AEs were thrombocytopenia (0.48, 95% CI: 0.36–0.59), neutropenia (0.37, 95% CI: 0.23–0.50), lymphopenia (0.33, 95% CI: 0.08–0.58) and anemia (0.16, 95% CI: 0.11–0.20). The most common non hematological AEs included diarrhea (0.14, 95% CI: 0.04–0.24), fatigue (0.12, 95% CI: 0.05–0.20), pneumonia (0.08, 95% CI: 0.03–0.13), nausea (0.04, 95% CI: 0.01–0.06) and so on. The results were shown in Fig. 4.

## Discussion

The findings of this systematic review and meta analyses demonstrated that panobinostat-based therapy was effective and generally tolerated well in patients with relapsed or/and refractory MM. The pooled and weighted ORR was 0.45, suggested that approximately 45% of the 700 patients displayed PR or better as the best response to panobinostat combined with other agents. And it should be highlighted that the ORR was 0.52 in the subgroup of panobinostat combining with Proteasome inhibitors (bertezomib or carfilzomib) with or without dexamethasone. The efficacy seemed to be enhanced by combining with proteasome inhibitors. Meanwhile, it seemed to be similar with the three-drug based regimen (panobinostat + bertezomib + dexamethasone) according to the ORR of 0.51.

Our findings have several clinical implications. First, the treatment for patients with MM is difficult and challenging once it has progressed. The regimen of panobinostat/bortezomib/dexamethasone for previously treated multiple myeloma has been approved by the newest NCCN Guidelines version 2.2016 for multiple myeloma^25^. Our meta analyses offered an additional proof to contribute to the newest NCCN. Second, though lack of phase III studies about panobinostat comparing with other first-line therapy agents or placebo, we can’t get the odd ratio compared to other agents or placebo. But according to the data from previous studies, we identified that a meta-analysis published by Kevin in 2014 showed an ORR of 39.1% (95% CI, 30.8–47.4%) for patients with relapsed/refractory myeloma under bortezomib-based retreatment^26^. Another meta-analysis about a novel agent pomalidomide after failure of lenalidomide and (or) bortezomib for multiple myeloma published by Zhixin Sheng in 2015 demonstrated that the ORR was 31% (95% CI: 19–50%) in the group of pomalidomide with low dose of dexamethasone^27^. Therefore panobinostat-based therapy for relapsed/refractory MM patients may be a better choice because of improving the ORR (45%, 95% CI: 31–59%).

Among the 11 trials, the only one phase III clinical trial demonstrated that the median time of PFS was 11.99months (95% CI: 10.33–12.94); The median time of TTP was 12.71months (95% CI: 11.3–14.06)^14^. Due to the varied length of follow-up time, the number of studies including PFS, TTP and OS is too small, there is no necessary to do meta analyses for these items.

Additionally, the safety of novel drugs used is also be focused on by clinicians. Our meta analyses showed that the most common Grade 3/4 AEs were hematologic. Different from previous studies, thrombocytopenia appeared to be outstanding among all the adverse effects with the rate of 0.48. The rate of neutropenia/lymphopenia/anemia was 0.37/0.33/0.16 separately. Therefore, clinicians should pay more attention to the hematological AEs. Besides, diarrhea/fatigue/pneumonia/nausea were common non-hematological AEs with the rates of 0.14/0.12/0.08/0.04. The incidence of thromboembolic events (VTE or PE) occurred great lower compared to IMiD. Peripheral neuropathies were main AEs under bortezomib therapy, the overall incidences of all- and high-grade peripheral neuropathies were 31.9% and 7.9% among MM patients^28^. Only two studies selected by our review mentioned peripheral neuropathies, both were combined with bertezomib and dexamethasone. Richardson’s trial showed the result that on-treatment peripheral neuropathy was mild, with only 1 patient having a grade >3 event which suggested that panobinostat could be safely combined with bortezomib without significant concern for increasing the severity of peripheral neuropathy. The phase III trial (San-Miguel) suggested that the frequency of peripheral neuropathy was similar with both treatments (Panobinostat plus bortezomib and dexamethasone versus placebo plus bortezomib and dexamethasone), and the frequency of grade 3–4 peripheral neuropathy for both treatment groups was similar to those reported in previous trials of intravenous bortezomib. Therefore, panobinostat would not increase the incidence of peripheral neuropathy. The data indicated that treatment with panobinostat combined with other agents appeared to be effective but didn’t increase the incidence of adverse events. To our knowledge, this is the first meta analyses focused on the novel drug panobinostat for the patients with relapsed or/and refractory MM.

However, the limitations in our meta analyses should be considered. First, the prognosis of patients is associated with the stage when they started under therapy, but most studies didn’t offer the exact data of each patient. Second, the treatment regimens were not the same between studies. Third, all of the selected studies were from Europe and America, only two of them included patients of Asian and only three included black/African American, so the analyses were considered as only for European and American populations. Furthermore, though all the selected studies were clinical trials, but most were single-arm studies, only one phase III study was included. It has been reported that three abnormalities t (4; 14), del (17p), and Amp (1q21) are considered as valuable predictors of poor outcome in MM patients treated with chemotherapy or stem cell transplantation^29^. Unfortunately, most studies offered only limited data of cytogenetic and FISH features of the patients.

## Conclusion

Treatment for relapsed or/and refractory MM patients with panobinostat combined with other agents appeared to be effective and generally well tolerated, with an ORR of 45%, especially combined with proteasome inhibitors. However, prolonged follow-up period is required to confirm the beneficial effects, more phase III clinical trials included large number of patients are warranted for further evaluation and help to establish the optimal dose and schedule.

## Additional Information

**How to cite this article**: Liu, J.- *et al.* Efficacy and Safety of Panobinostat in Relapsed or/and Refractory Multiple Myeloma: Meta Analyses of Clinical Trials and Systematic Review. *Sci. Rep.* **6**, 27361; doi: 10.1038/srep27361 (2016).

## Figures and Tables

**Table 1:** Characteristics of the studies about panobinostat combined with other agents for patients with relapsed/refractory multiple myeloma.

Study	Year	Study design	Total No.	Female/Male (n)	Mean age (y)	ISS stage:1/2/3/unknown	Dose of panobinostat	Combined agents	ORR
Chari	2015	phase II	20	–	64	–	20 mg	Len&DXM	0.45
Berdeja	2015	phase I/II	42	25/17	66	20/14/5/5	20–30 mg	CFZ	0.67
San-Miguel	2014	phase III	387	185/202	63	156/104/77/50	20 mg	BTZ&DXM	0.61
Mangiacavalli	2014	phase II	15	–	–	–	20 mg	BTZ&DXM	0.67
Kaufman	2014	phase I	20	–	64.5	–	15–20 mg	CFZ	0.50
Berenson	2014	phase I/II	40	15/25	65.4	–	10–20 mg	Mel	0.09
San-Miguel	2013	phase Ib	62	19/43	62	25/15/20/2	10–20 mg	BTZ&DXM	0.52
Richardson	2013	phase II	55	26/29	61	18/23/13/1	20 mg	BTZ&DXM	0.35
Zangari	2012	phase I	11	–	58	–	5–15 mg	BTZ	0.36
Shah	2012	phase Ib	17	6/11	62	–	15 mg	CFZ	0.35
Offidani	2012	phase II	31	15/16	68	17/10/4/0	10–20 mg	Mel&PED&Tha	0.39

ISS, International Staging System; Len, lenalidomide; BTZ, bortezomib; DXM, dexamethasone; CFZ, carfilzomib; Mel, melphalan; PED, prednisone; Tha, thalidomide.

**Table 2:**
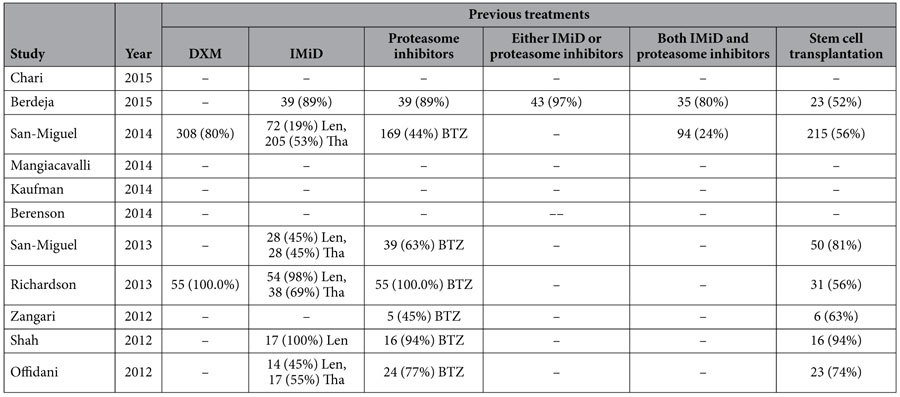
Previous treatments of the studies about panobinostat combined with other agents for patients with relapsed/refractory multiple myeloma.
